# The impact of work–family conflict on occupational fatigue among endoscopy nurses in China: a moderated mediation model

**DOI:** 10.3389/fpubh.2024.1485143

**Published:** 2024-10-23

**Authors:** Zhi Zeng, Sumei Zhou, Meng Liu, Guiqiong Xie, Yazhi He, Jiquan Zhang

**Affiliations:** ^1^Department of Gastroenterology, Deyang People’s Hospital, Deyang, Sichuan, China; ^2^Department of Neurosurgery, Deyang People’s Hospital, Deyang, Sichuan, China; ^3^Pediatric Ward 2 (Children’s Blood/Cancer Ward), Sichuan Provincial People’s Hospital, Chengdu, China; ^4^Department of Nephrology, Deyang People’s Hospital, Deyang, Sichuan, China

**Keywords:** endoscopy nurses, occupational fatigue, work–family conflict, positive coping style, perceived social support, moderating mediation

## Abstract

**Background:**

Endoscopy nurses in China face significant work–family conflicts, where the clash between high work demands and family responsibilities markedly increases the risk of occupational fatigue. This not only affects the nurses’ physical and mental health and overall well-being, but also poses a threat to the quality of care and patient safety. This study, grounded in the Conservation of Resources theory, constructs a moderated mediation model to examine the mediating role of positive coping style in the relationship between work–family conflict and occupational fatigue among endoscopy nurses in China, as well as the moderating effect of perceived social support.

**Methods:**

A convenience sampling method was employed to select 315 endoscopy nurses from 25 tertiary hospitals across 14 provinces in China. A questionnaire survey was conducted using the Fatigue Assessment Instrument, the Work–Family Conflict Scale, the Simplified Coping Style Questionnaire, and the Perceived Social Support Scale. The moderated mediation model was validated using Stata16.0.

**Results:**

Our findings reveal that work–family conflict is a significant predictor of occupational fatigue, with a negative impact on positive coping style. Positive coping style, in turn, is negatively associated with occupational fatigue. Furthermore, positive coping style partially mediates the relationship between work–family conflict and occupational fatigue, accounting for 35.52% of the total effect. Additionally, perceived social support mitigates the negative effects of work–family conflict on positive coping style and occupational fatigue.

**Conclusion:**

There exists a moderated mediation effect between work–family conflict and occupational fatigue among endoscopy nurses in China, wherein positive coping style serve as a mediating variable. Perceived social support mitigates the negative impact of work–family conflict on positive coping style, while enhancing the alleviating effect of positive coping style on occupational fatigue. Therefore, improving endoscopy nurses’ levels of perceived social support and coping strategies may help to prevent and alleviate the occurrence of occupational fatigue.

## 1 Introduction

By the end of 2023, the total number of registered nurses in China had reached 5.63 million (1), playing a crucial role in maintaining public health and providing high-quality medical services. However, with the continuous growth in demand for medical services and the significant increase in workload, nurses are experiencing escalating work-related stress, leading to growing concerns about their physical and mental well-being (2, 3). This issue has become a pressing matter requiring urgent attention.

Occupational fatigue is often considered a major indicator of an unhealthy lifestyle and is defined as a syndrome where an individual’s physical and mental health, as well as their normal life, are disrupted due to prolonged and intense work (4). Research has demonstrated that fatigue among nurses impairs cognitive functions, resulting in prolonged reaction times, diminished alertness, and distortions in perception and cognition. This, in turn, leads to reduced memory, impaired information processing, decreased attention, and motivation, thus increasing the safety risks in clinical practice (5). For endoscopy nurses, the high-intensity work environment, which demands sustained focus and meticulous operation over extended periods coupled with irregular working hours, exacerbates the issue of occupational fatigue (6). According to the Conservation of Resources (COR) theory (7), occupational fatigue represents a significant depletion of individual resources, which in turn escalates stress and diminishes the ability to cope with it. The prevalence of occupational fatigue among endoscopy nurses is reported to be as high as 57.32% (6, 8), indicating that fatigue has markedly impaired their capacity to manage the high-pressure demands of endoscopic procedures, including declines in operational skills and judgment. Furthermore, occupational fatigue may lead to health issues such as cervical spondylosis and sleep disorders, which further compromise work efficiency and increase the risk of errors, thereby adversely affecting patient treatment outcomes and safety (9, 10). Therefore, addressing occupational fatigue is crucial for safeguarding the health of endoscopy nurses, enhancing the quality of care, and ensuring patient safety.

### 1.1 Work–family conflict and occupational fatigue

Work–family conflict refers to the clash between demands and responsibilities associated with work and family roles, making it challenging for individuals to balance these two roles (11, 12). Research indicates that endoscopy nurses frequently encounter difficulties in balancing work and family roles, with approximately 50% of nurses experiencing long-term conflicts between work and family life (13). This conflict not only negatively impacts individual mental health but may also affect patient safety (14, 15). According to the Interactionist Model (16), high levels of work–family conflict increase both psychological and physical burdens on individuals, leading to emotional exhaustion, symptoms of depression, and decreased well-being, thereby heightening the risk of occupational fatigue. The COR theory posits that occupational fatigue arises from a state of physical and mental exhaustion due to resource depletion (4, 9, 17). Work–family conflict exacerbates occupational fatigue by depleting key resources such as time, energy, and emotional support. For endoscopy nurses, work–family conflict not only results in the depletion of resources between work and family roles but also further undermines their ability to cope with stress, thereby intensifying occupational fatigue. Hence, work–family conflict is a significant factor contributing to occupational fatigue. Based on these considerations, we propose the following hypothesis:

*Hypothesis 1 (H1)*: There is a positive correlation between work–family conflict and occupational fatigue among endoscopy nurses.

### 1.2 The mediating role of positive coping style

Positive coping style refers to an individual’s approach to handling stress events by actively adjusting their cognition and behavior, adopting a proactive attitude, and employing strategies to adapt to their environment (18). Existing research indicates that coping styles play a crucial role in moderating the impact of stress and setbacks (6, 19). For endoscopy nurses, a positive coping style not only effectively alleviates high-intensity work pressure but also improves emotional states and manages work–family conflicts effectively, thereby reducing the risk of occupational fatigue and enhancing work efficiency and psychological well-being (20). The COR theory suggests that individuals (21), when facing stress and conflict, strive to protect and accumulate resources to cope with and mitigate stress. Stress induced by work–family conflict affects endoscopy nurses’ resource use, which in turn influences their choice of coping style. Previous studies have shown that positive coping style is negatively correlated with work–family conflict (22). Specifically, employing a more positive coping style helps reduce the negative impact of work–family conflict, thereby improving psychological health and occupational efficiency. Based on these considerations, we propose the following hypothesis:

*Hypothesis 2 (H2)*: Positive coping style mediates the relationship between work–family conflict and occupational fatigue among endoscopy nurses.

### 1.3 The moderating role of perceived social support

Perceived social support, which refers to an individual’s subjective perception of understanding and help from colleagues, family, and friends, is a crucial psychological resource (23). According to the COR theory (24), social support can enhance endoscopy nurses’ resource reserves, providing them with more resources to effectively cope with stress when facing work–family conflicts. When endoscopy nurses perceive higher levels of social support, their sense of psychological safety is enhanced, leading them to be more likely to adopt positive coping style. This positive attitude helps mitigate the negative impact of work–family conflict, thereby improving the nurses’ job performance and quality of life (25). This suggests that perceived social support may play a moderating role between work–family conflict and positive coping style.

Social support theory further indicates (26) that external support can help individuals alleviate work stress, enhance psychological resilience, and promote physical and mental health. Research by Slobodskaya and Kornienko (27) shows a positive correlation between perceived social support and positive coping style, meaning that when nurses perceive social support, they are more likely to use positive coping style to manage work stress. Additionally, nurses who feel supported are better able to manage stress and interpersonal relationships, maintain a positive attitude, and thereby reduce the risk of occupational fatigue (28). In contrast, nurses who lack perceived social support are more likely to feel isolated and stressed, which can exacerbate occupational fatigue. For endoscopy nurses, high levels of social support are undoubtedly a valuable emotional resource that enhances their confidence and coping ability, helping them manage work stress more effectively and thereby reducing the likelihood of occupational fatigue (8, 25, 29). Conversely, a lack of social support can lead to deeper feelings of isolation and increased stress, significantly worsening their occupational fatigue. Based on this analysis, we propose the following hypothesis:

*Hypothesis 3 (H3)*: Perceived social support moderates the impact of work–family conflict on positive coping style, and it also moderates the effect of positive coping style on occupational fatigue among endoscopy nurses.

**Figure 1:**
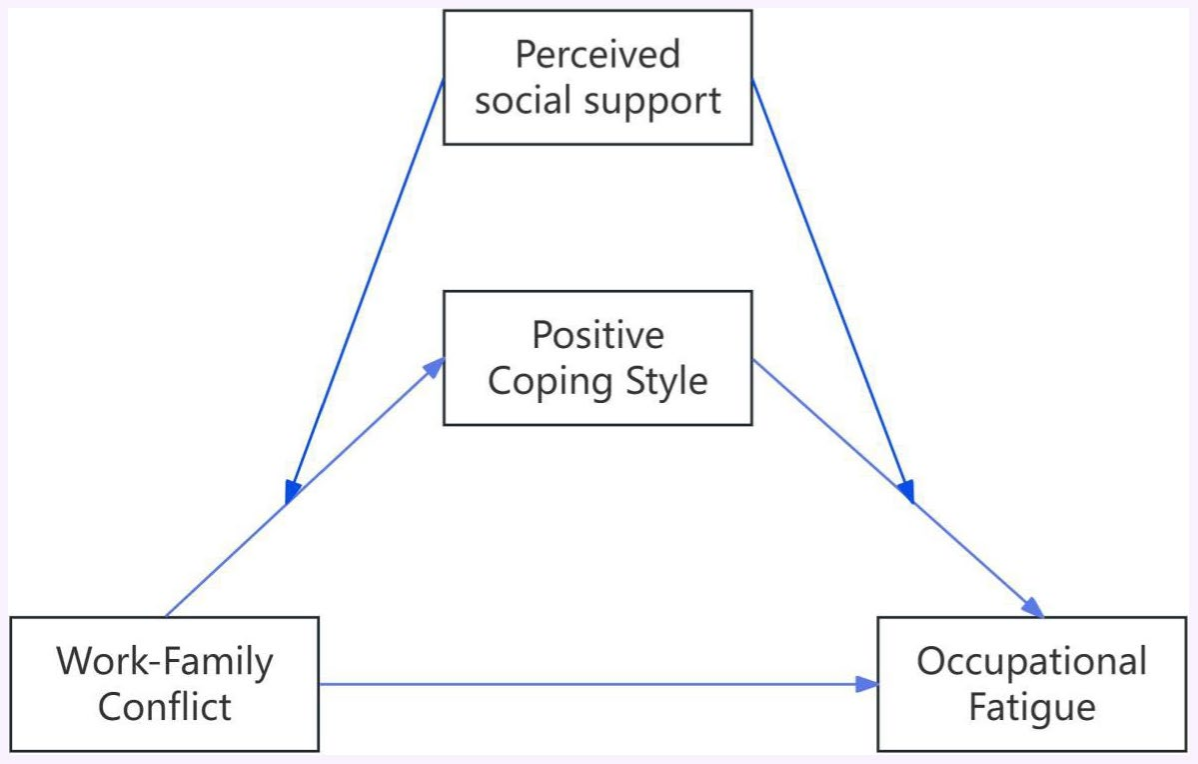
The proposed mediated moderation model.

Based on the Conservation of Resources theory, this study constructs a moderated mediation model (Figure 1) to delve into the intrinsic relationship between work–family conflict and occupational fatigue among endoscopy nurses. We aim to effectively enhance the work experience and physical and mental health of endoscopy nurses by optimizing coping strategies and strengthening perceived social support, providing a theoretical basis for preventing and improving occupational fatigue among them.

## 2 Materials and methods

### 2.1 Study design and participants

This study employed a cross-sectional design and selected endoscopy nurses from 25 hospitals across 14 provinces in China, including Zhejiang, Sichuan, Henan, Anhui, Shandong, Hubei, Jiangsu, Guangdong, and Chongqing, as the study population. Data collection was conducted from July 1 to July 31, 2024.

Inclusion criteria: nurses with at least 2 years of experience in endoscopy nursing; providing informed consent and voluntarily participating in the survey.

Exclusion criteria: nurses who were undergoing further education or were retired and re-employed during the study period; nurses who were not on duty due to travel, leave, or other special circumstances. Figure 1 showed the process of participant selection.

**Figure 2:**
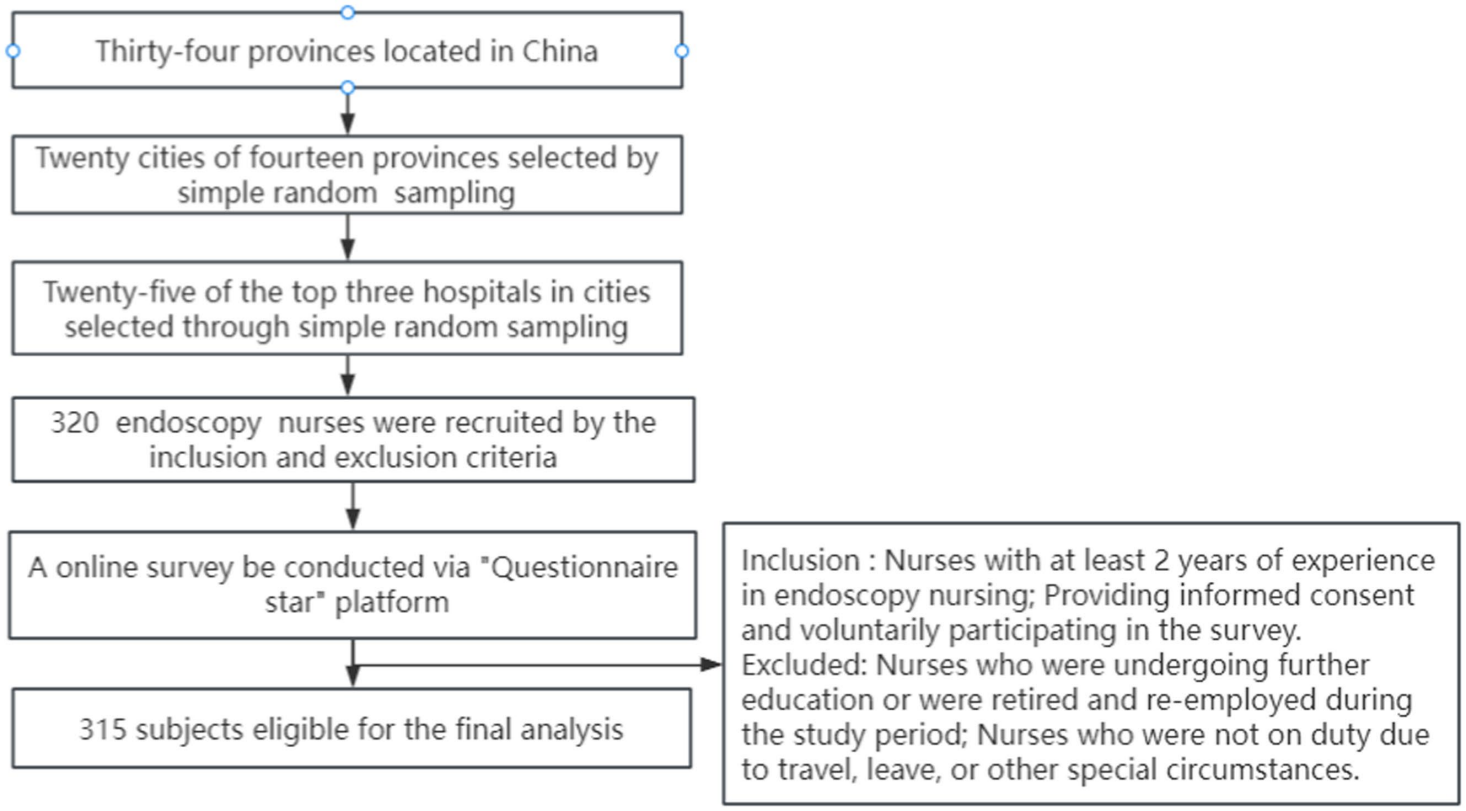
The process of participant selection.

Based on the sample size requirements for mediation analysis and the standards proposed by Pan et al. (30), a sample size of 218 is needed to achieve an 80% power level with a medium effect size, calculated using the Bootstrap method. To account for an anticipated 20% rate of incomplete or invalid questionnaires, a final sample size of 262 valid responses was required. A total of 323 questionnaires were distributed, with 320 being returned. After excluding 5 invalid questionnaires due to data missing, evident patterns, or logical inconsistencies, 315 valid responses were obtained, resulting in an effective response rate of 98.40% (Figure 2).

### 2.2 Ethical considerations

The study approved by the Ethics Committee of Deyang people’s Hospital, and the personal information of participants was anonymously treated for privacy and confidentiality.

### 2.3 Research tools

#### 2.3.1 Demographic variables

These were self-designed by the research team and included gender, age, marital status, fertility status, educational level, professional rank, years of work, daily working hours, weekly working days, and monthly income.

#### 2.3.2 Fatigue assessment instrument

Occupational fatigue was assessed using the Fatigue Assessment Instrument (FAI). The FAI was developed by Schwartz et al. (31), and revised by Wang et al. (32) for a Chinese version. The instrument comprises four dimensions: the severity of fatigue, the specific aspects of the environment that contribute to fatigue, the consequences of fatigue, and the response of fatigue to rest and sleep, with a total of 29 items. Each item is scored on a 7-point Likert scale (1 indicating “strongly disagree” and 7 indicating “strongly agree”), with a total score range of 4–28. The FAI score is determined by calculating the average score of each dimension, with each dimension’s score being the average of the ratings for all items within that dimension. The sum of the scores across all dimensions is the total score, with a higher score indicating a higher degree of fatigue. In previous studies, the Cronbach’s *α* coefficient for the FAI was 0.768–0.916 (33), while in the current study, the Cronbach’s *α* coefficient was 0.851.

#### 2.3.3 Work–family conflict

Work–family conflict was assessed using the Work–Family Conflict Scale (WFC). The WFC was developed by Carlson et al. (34), and translated and revised into Chinese by Bai et al. (35). The scale includes two dimensions: work-to-family conflict and family-to-work conflict, with a total of 18 items. The responses are rated on a 5-point Likert scale (1 indicating “strongly disagree” and 5 indicating “strongly agree”), with a total score range of 18–90. Higher scores indicate a higher level of conflict between work and family. Prior studies have demonstrated high reliability and validity for the WFC among Chinese nurses, with a Cronbach’s *α* coefficient of 0.868 (36). In the current study, the Cronbach’s *α* coefficient was 0.863.

#### 2.3.4 Positive coping style

Positive coping style were measured using the Simple Coping Style Questionnaire (SCSQ). The SCSQ was designed by Folkman and Lazarus (37) and adapted into Chinese by Xie (38). The questionnaire consists of two dimensions: positive coping and negative coping, with a total of 20 items. For this study, only the positive coping dimension was used to evaluate the positive coping skills of endoscopy nurses, including 12 items. The scale employs a 4-point Likert scale (0 indicating “not used” and 3 indicating “often used”). The total score ranges from 0 to 36, with higher scores reflecting a greater tendency to employ positive coping style. The positive coping dimension of the SCSQ has demonstrated good reliability and validity, with a Cronbach’s *α* coefficient of 0.810 (39). In this study, the Cronbach’s *α* coefficient was 0.910.

#### 2.3.5 Perceived social support

This study assessed the level of perceived social support using the Perceived Social Support Scale (PSSS). The PSSS was developed by Zimet et al. (40) and later translated and adapted into Chinese by Jia and Yue (41). The scale is a self-report questionnaire comprising three dimensions: family support, friend support, and support from significant others, with a total of 12 items. Responses are rated on a 7-point Likert scale (1 indicating “strongly disagree” and 7 indicating “strongly agree”), with a total score range from 12 to 84. Higher scores reflect a greater level of perceived social support. The PSSS has demonstrated high reliability and validity (42), with a Cronbach’s *α* coefficient of 0.957. In this study, the Cronbach’s *α* coefficient was 0.952.

### 2.4 Data collection

Prior to data collection, the research team contacted the head nurses of endoscopy units at each hospital to provide a detailed explanation of the study’s objectives, significance, and procedures, and to request their assistance in distributing the questionnaires. Following this, the team asked the endoscopy nurses if they were willing to participate in the survey. Upon receiving their consent, the electronic survey link was distributed via WeChat through the Wenjuanxing online platform.

To ensure data validity, all questions were set as mandatory, with restrictions on submissions from the same IP address or WeChat account to prevent multiple responses, and a minimum completion time of 10 min was enforced. After the survey concluded, the research team reviewed and downloaded the data, excluding any questionnaires that exhibited obvious patterns or logical inconsistencies.

### 2.5 Statistical analyses

Data analysis was conducted using Stata16.0 software. Categorical data were presented as frequencies or percentages, while continuous data were reported as means ± standard deviations (M ± SD). To examine common method bias, Harman’s single-factor test was performed. The reliability and validity of the scales were assessed using the Kaiser–Meyer–Olkin (KMO) measure and Bartlett’s test of sphericity. Pearson correlation coefficients were used to explore the linear relationships among occupational fatigue, work–family conflict, perceived social support, and positive coping style.

A mediation effect model was employed to test whether positive coping plays a mediating role between work–family conflict and occupational fatigue. A moderated mediation model was used to assess the moderating effect of perceived social support on the relationship between work–family conflict and occupational fatigue. Indirect effects were evaluated using the Bootstrapping method (*n* = 5,000) with 95% confidence intervals; a significant moderated mediation effect was indicated if the 95%CI did not include zero (43). Finally, simple slope plots were generated to visualize the impact of perceived social support levels on the interaction effects. The significance level was set at *p* < 0.05.

## 3 Results

### 3.1 General demographic information

A total of 315 valid questionnaires were collected, and the survey results are shown in Table 1.

**Table 1:** General demographic information.

Variable	Category	Number	Percentage (%)
Gender	Males	71	22.54
	Females	244	77.46
Age	Mean age	37.29 ± 8.22	–
	Age range	20 ~ 58	–
Marital status	Single	35	11.11
	Married	273	86.67
	Divorced	7	2.22
Fertility status	Childless	15	4.76
	One child	72	22.86
	Two children	222	70.18
	More than two children	6	1.9
Education level	Associate degree	26	8.25
	Bachelor degree	260	82.54
	Master degree	29	9.21
Professional title	Junior title	206	65.4
	Intermediate title	86	27.3
	Senior title	23	7.3
Years of work	Less than 3 years	238	75.55
	3 ~ 5 years	24	7.62
	5 ~ 10 years	32	10.16
	More than 10 years	21	6.67
Daily work hours	Less than 8 h	34	10.8
	8 ~ 9 h	77	24.44
	9 ~ 10 h	70	22.22
	10 ~ 12 h	86	27.3
	More than 12 h	48	15.24
Weekly work days	5 days	116	36.82
	5 ~ 5.5 days	118	37.46
	5.5 ~ 6 days	53	16.83
	6 ~ 6.5 days	20	6.35
	6.5 ~ 7 days	8	2.54
Monthly income	Less than 5,000 yuan	5	1.59
	5,000 ~ 7,000 yuan	25	7.94
	7,000 ~ 9,000 yuan	89	28.25
	9,000 ~ 12,000 yuan	153	48.57
	More than 12,000 yuan	43	13.65

### 3.2 Common method bias test

The results of Harman’s single-factor test revealed that the first factor explained 15.67% of the variance, which is below the 40% threshold. This indicates that the study does not suffer from significant common method bias.

### 3.3 Scale reliability and validity

In this study, the Cronbach’s alpha coefficients for all scales were greater than 0.85, indicating excellent internal consistency and high reliability. The KMO test results showed that all KMO values exceeded 0.75. Additionally, Bartlett’s test of sphericity yielded *p*-values of 0.01, further supporting the scales’ good validity. Detailed parameters are provided in Table 2.

**Table 2:** Reliability and validity testing results of the scale.

Variables	Cronbach’s *α*	KMO	Bartlett’s test of sphericity (*P*)
Occupational fatigue	0.863	0.835	3654.462 (0.000)
Work–family conflict	0.851	0.782	3987.613 (0.000)
Positive coping style	0.910	0.889	2411.563 (0.000)
Perceived social support	0.952	0.916	5272.385 (0.000)

### 3.4 Descriptive statistics and correlation analysis of variables

Descriptive statistics and correlation analysis are presented in Table 3. The study found a significant positive correlation between occupational fatigue and work–family conflict (*r* = 0.207, *p* < 0.001). In contrast, occupational fatigue was significantly negatively correlated with positive coping style (*r* = −0.354, *p* < 0.001) and perceived social support (*r* = −0.292, *p* < 0.001). Work–family conflict was significantly negatively correlated with positive coping style (*r* = −0.250, *p* < 0.001) and perceived social support (*r* = −0.101, *p* < 0.05). Additionally, positive coping was significantly positively correlated with perceived social support (*r* = 0.218, *p* < 0.001).

**Table 3:** Descriptive statistics and Pearson correlations between study variables.

Variables	M ± SD	1	2	3	4
1 Occupational fatigue	22.099 ± 2.608	1			
2 Work–family conflict	55.222 ± 10.406	0.207***	1		
3 Positive coping style	22.337 ± 6.825	−0.354***	−0.250***	1	
4 Perceived social support	57.771 ± 17.078	−0.292***	−0.101*	0.218***	1

*P < 0.05, **P < 0.01, ***P < 0.001.

### 3.5 Testing the mediating effect of positive coping style

The results of the mediation model for positive coping style are shown in Table 4. The findings indicate that work–family conflict significantly positively predicts occupational fatigue (*β* = 0.051, *p* < 0.001), confirming Hypothesis 1. Additionally, work–family conflict significantly negatively predicts positive coping style (*β* = −0.140, *p* < 0.001). When work–family conflict is tested as a predictor of occupational fatigue alongside positive coping style, work–family conflict still significantly positively predicts occupational fatigue (*β* = 0.033, *p* < 0.001), while positive coping style significantly negatively predict occupational fatigue (*β* = −0.129, *p* < 0.001).

**Table 4:** Regression results of mediating effect of positive coping style.

Dependent variable	Independent variable	*β*	SE	*t*	*P*	*R* ^2^	*F*
Occupational fatigue	Work–family conflict	0.051	0.014	3.57	0.000	0.0718	1.79
Positive coping style	Work–family conflict	−0.140	0.036	−3.92	0.000	0.1485	4.04
Occupational fatigue	Work–family conflict	0.033	0.014	2.37	0.018	0.1692	4.37
	Positive coping style	−0.129	0.022	−5.93	0.000		

β, values are standardized coefficients.

Bootstrap analysis further indicated that the direct effect of work–family conflict on occupational fatigue was 0.033, with a 95%CI that did not include zero, suggesting that the direct effect of work–family conflict on occupational fatigue is significant. The indirect effect was 0.018, with a 95%CI that also did not include zero, indicating that the indirect effect of work–family conflict on occupational fatigue is significant. Therefore, positive coping style play a partial mediating role between work–family conflict and occupational fatigue, with the mediating effect accounting for 35.52% of the total effect. This supports Hypothesis 2, with detailed parameters provided in Table 5.

**Table 5:** Mediating effects of positive coping style.

Project	Standardized effect value	Relative effect value	95%CI	*P*
Direct effect	0.033	64.48%	0.001–0.065	0.000
Indirect effect	0.018	35.52%	0.008–0.028	0.000
Total effect	0.051	100%	0.019–0.083	0.000

### 3.6 Testing for the moderated mediation model

The regression results for the moderating effect of perceived social support on the mediation effect of positive coping style are presented in Table 6. The results indicate a significant positive correlation between work–family conflict and occupational fatigue (*β* = 0.055, *p* < 0.01). Additionally, the interactions between perceived social support and work–family conflict, as well as perceived social support and positive coping style, had significant negative impacts on occupational fatigue (*β* = −0.001, *p* < 0.01; *β* = −0.001, *p* < 0.05), demonstrating that perceived social support plays a moderating role in the relationship between work–family conflict and occupational fatigue, thus supporting Hypothesis 3.

**Table 6:** Regression results of the moderating effect of social support on the mediated relationship of positive coping style.

Dependent variable	Independent variable	*β*	SE	*t*	*P*	*R* ^2^	*F*
Occupational fatigue	Work–family conflict	0.055	0.016	3.38	0.001	0.269	7.80
	Positive coping style	−0.055	0.045	−1.21	0.227		
	Perceived social support	−0.371	1.435	−0.26	0.796		
	Work–family conflict × Perceived social support	−0.001	0.0003	−2.6	0.01		
	Positive coping style × Perceived social support	−0.001	0.001	−2.08	0.038		

**Figure 3:**
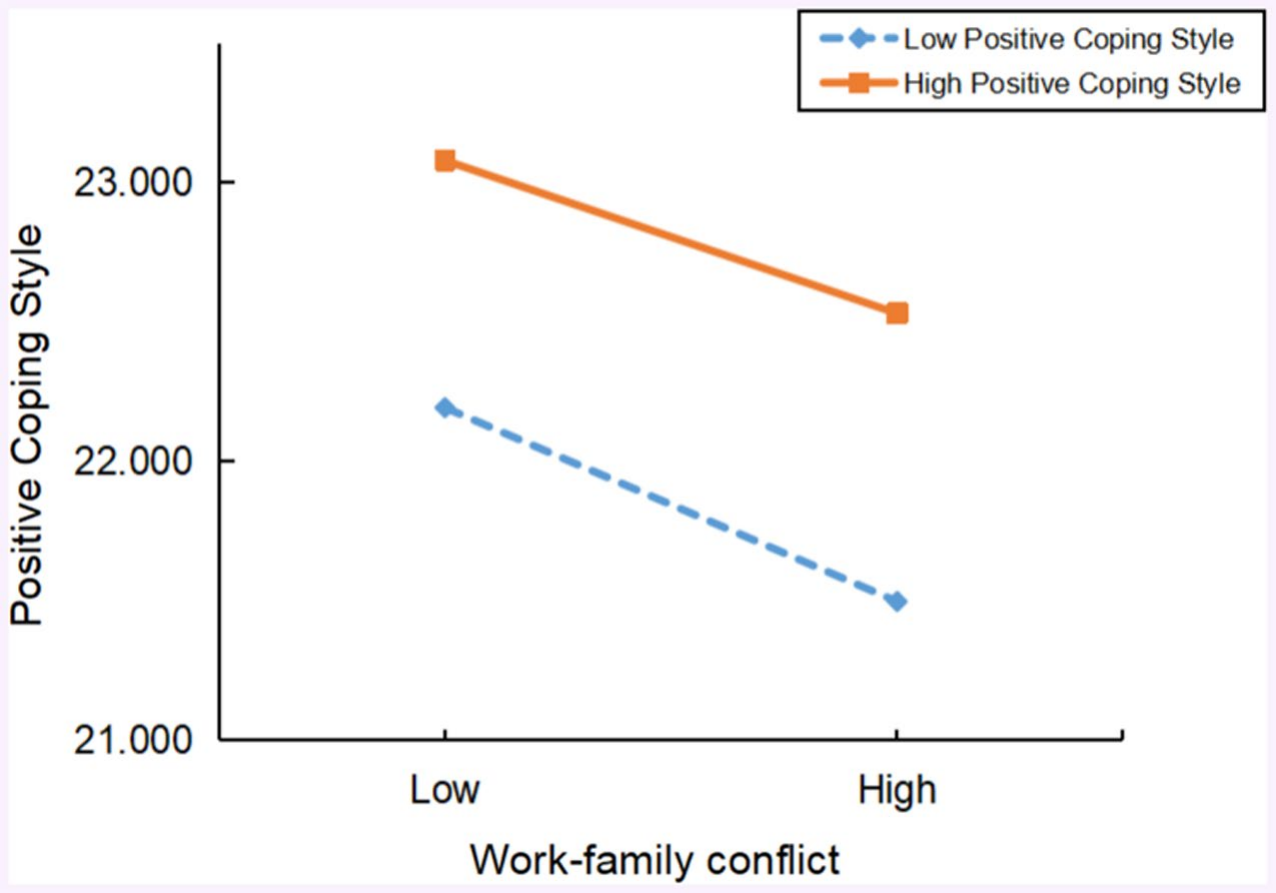
The moderating effect of social support on the relationship between work–family conflict and positive coping style.

To further explore the moderating effect of perceived social support, the sample was divided into high (*Z* ≥ 1*SD*) and low (*Z* ≤ −1*SD*) groups based on standard scores of perceived social support for simple slope analysis. Figure 3 shows that, at a low level of perceived social support, the negative predictive effect of work–family conflict on positive coping style (Bsimple = −0.154, *t* = −2.220, *p* < 0.05) was significantly higher than at a high level of perceived social support (Bsimple = −0.074, *t* = −2.750, *p* < 0.05), confirming that perceived social support moderates the early part of the mediation path of positive coping style in the relationship between work–family conflict and occupational fatigue.

**Figure 4:**
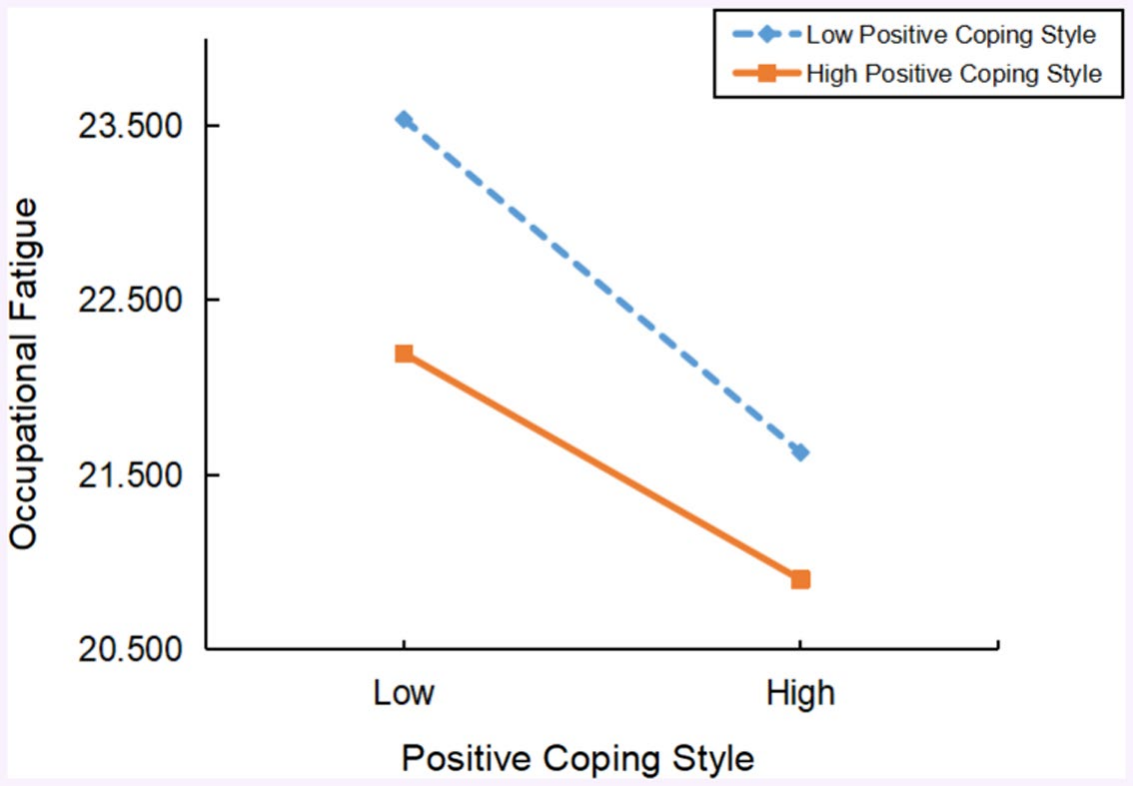
The moderating effect of social support on the relationship between positive coping and occupational fatigue.

Figure 4 further indicates that, at a high level of perceived social support, higher positive coping style is associated with more significant improvement in occupational fatigue (Bsimple = −0.114, *t* = −2.100, *p* < 0.05). Similarly, at a low level of perceived social support, an increase in positive coping style also significantly improves occupational fatigue (Bsimple = −0.197, *t* = −4.060, *p* < 0.05). This further verifies the moderating role of perceived social support in the later part of the mediation path of positive coping style in the relationship between work–family conflict and occupational fatigue.

## 4 Discussion

### 4.1 The impact of work–family conflict on occupational fatigue

This study reveals that work–family conflict significantly predicts occupational fatigue among endoscopy nurses, indicating that as conflicts between work and family roles intensify, so does occupational fatigue. These findings are consistent with the research results of Li et al. (44) and Chen et al. (45). According to the COR theory (46), the “resource loss” process suggests that time and energy are finite resources. When these resources are excessively depleted through demanding work and are not adequately replenished, work stress leads to resource loss. This, in turn, impairs an individual’s ability to fulfill family responsibilities, exacerbating work–family conflict and increasing the risk of occupational fatigue. In high-intensity work environments, endoscopy nurses often expend significant amounts of time and psychological resources, which can lead to neglect of family roles, making them more vulnerable to balancing work demands and family responsibilities and thus intensifying occupational fatigue (47).

Moreover, work–family conflict significantly increases the emotional burden on endoscopy nurses, leading to negative emotions such as anxiety, exhaustion, and depression. The accumulation of these negative emotions over time impairs their ability to maintain a positive psychological state, further aggravating occupational fatigue (45). Additionally, prolonged work–family conflict can result in physiological discomforts, such as sleep disturbances and reduced immunity, which further intensify occupational fatigue (17). Therefore, work–family conflict affects occupational fatigue among endoscopy nurses through various mechanisms, including the depletion of psychological resources, increased emotional burden, and physiological health issues.

It is recommended that nursing managers implement flexible scheduling systems, provide mental health support, and establish family-centered welfare activities, such as family team-building events, to help nurses better balance work and family life and reduce the incidence of occupational fatigue.

### 4.2 The mediating role of positive coping style

The current study found that positive coping style played a partial mediating role between work–family conflict and occupational fatigue among endoscopy nurses. According to the COR theory’s “resource gain” process (21), individuals facing work–family conflict can acquire additional positive psychological resources (such as positive emotions, motivation, and inspiration) through the adoption of positive coping style, thereby mitigating the negative impacts of conflict and alleviating occupational fatigue. Positive coping style act as a protective mechanism of psychological resources, effectively preventing the excessive depletion of such resources and providing a “buffer zone” for nurses in long-term high-stress work environments to mitigate the negative impacts of conflict (48, 49). In positive coping style, emotional regulation plays a crucial role (50, 51). By effectively managing emotions, endoscopy nurses can reduce their immediate emotional burden and establish a positive emotional cycle, which enhances their ability to cope with future conflicts and contributes to maintaining mental health and alleviating occupational fatigue (52, 53). Additionally, positive coping style can also significantly enhance the professional identity of endoscopy nurses (6). By successfully navigating work and family conflicts and challenges, nurses can experience the realization of their self-worth and an increase in professional fulfillment, thereby enhancing job satisfaction and intrinsic motivation, allowing them to better cope with future challenges.

Therefore, this suggests that managers should focus on the positive coping style of endoscopy nurses by reinforcing training in emotional regulation, providing psychological support services, optimizing work arrangements, enhancing professional identity, and strengthening family support to assist nurses in effectively coping with work–family conflict, preserving psychological resources, and alleviating occupational fatigue.

### 4.3 The moderating role of perceived social support

The results of this study indicate that perceived social support moderates both the initial and subsequent stages of the mediation path involving “work–family conflict—positive coping style—occupational fatigue.” Specifically, high levels of perceived social support have a significant protective effect on endoscopy nurses, effectively mitigating the potential impact of work–family conflict on occupational fatigue. This finding partially supports the COR theory (7). When faced with stressors, social support from colleagues, friends, and family serves as a unique personal resource that is crucial for reducing stress levels among nurses.

In the context of endoscopy nurses, work–family conflict is a prominent stressor that can profoundly affect psychological well-being and job performance (54). Perceived social support, as an important protective resource, plays a critical role in alleviating stress, enhancing coping abilities, and fostering a positive psychological state (55). Specifically, endoscopy nurses with high levels of perceived social support are better able to employ positive coping styles such as emotional regulation and problem-solving when dealing with work–family conflict, thereby mitigating the negative impacts of such conflict (56, 57). Additionally, emotional support, assistance, and encouragement from family, colleagues, or organizations can enhance nurses’ self-efficacy and coping capacity, further weakening the positive predictive effect of conflict on occupational fatigue. Conversely, nurses lacking perceived social support often feel helpless and frustrated when facing work–family conflict, lacking effective positive coping styles, and are more prone to falling into negative emotional cycles, which exacerbates occupational fatigue.

Overall, the moderating effect of perceived social support manifests at multiple levels: (i) Enhancement of Emotional Support: High levels of perceived social support provide emotional comfort from colleagues, friends, and family, increasing endoscopy nurses’ confidence in handling work–family conflict and making them more inclined to adopt positive coping styles, thus reducing occupational fatigue (29, 55). (ii) Effective Utilization of Resources: Perceived social support encourages endoscopy nurses to actively seek help at work and build support networks, thereby improving work efficiency and reducing occupational fatigue caused by excessive psychological stress (58). (iii) Promotion of Positive Psychological State: Perceived social support helps endoscopy nurses maintain positive emotions and psychological states, enhancing work motivation and engagement, and lowering the risk of occupational fatigue (59, 60). (iv) Optimization of Coping Strategies: Perceived social support facilitates the adoption of effective coping strategies, which not only alleviate current stress but also improve overall job adaptability and reduce the risk of occupational fatigue (61).

Therefore, it is recommended that nursing managers prioritize and optimize the perceived social support network for nurses. By enhancing nurses’ perception and utilization of social support, managers can help them better manage work–family conflicts, thereby increasing job satisfaction, maintaining psychological health, and effectively alleviating the impact of occupational fatigue.

## 5 Study limitations

This study explores the mediating role of positive coping styles in the relationship between work–family conflict and occupational fatigue among endoscopy nurses, as well as the moderating mechanism of perceived social support. However, there are several limitations to this research. Firstly, the study primarily relies on self-report scales to measure variables such as work–family conflict, perceived social support, and occupational fatigue. Despite implementing quality control measures such as anonymous responses, controlling survey timing, and data cleaning, self-report scales may still be subject to subjective biases and consistency biases, potentially leading to common method bias. To enhance the validity of future research, it is recommended to incorporate multiple measurement methods, such as behavioral observations and physiological indicators, to complement the limitations of self-report scales. Secondly, the study employed a cross-sectional design, which does not fully reveal the causal relationships between variables. Future research should utilize longitudinal designs for long-term tracking to explore the dynamic relationships between these variables, thus providing deeper causal evidence and effective intervention strategies. Furthermore, as the participants in this study were all Chinese endoscopic nurses, the findings may be influenced by the unique cultural characteristics of China, such as collectivism and a strong emphasis on family. These cultural factors may shape the way nurses achieve a balance between work and family, thereby affecting their experiences of work–family conflict and occupational fatigue. Additionally, nurses in other countries or different departments may face distinct types of work–family conflicts and adopt different coping strategies in response. Therefore, to achieve a more comprehensive understanding, it is recommended that future research incorporates diverse cultural and occupational groups to explore the impact of work–family conflict on occupational fatigue, with the aim of deriving broader and more applicable conclusions.

## 6 Conclusion

The results of this study indicate that: (i) Work–family conflict has a significant positive predictive effect on occupational fatigue among endoscopy nurses, meaning that an imbalance between work and family life leads to increased occupational fatigue. (ii) Positive coping styles play a partial mediating role in the relationship between work–family conflict and occupational fatigue among endoscopy nurses. (iii) Perceived social support moderates the mediating path of positive coping styles, meaning that high levels of perceived social support not only weaken the impact of work–family conflict on positive coping styles but also enhance the effectiveness of positive coping styles in reducing occupational fatigue.

In summary, this study reveals that enhancing endoscopy nurses’ perceived social support levels and positive coping styles can effectively reduce the positive effect of work–family conflict on occupational fatigue. This provides an important theoretical basis for preventing and improving occupational fatigue among endoscopy nurses and contributes to improving the physical and mental health levels of the nursing team.

## Data Availability

The original contributions presented in the study are included in the article/supplementary material, further inquiries can be directed to the corresponding author.
